# Editorial: Engineering probiotics for multiple interventions on intestinal diseases

**DOI:** 10.3389/fcimb.2023.1138998

**Published:** 2023-01-20

**Authors:** He Huang, Huabing Yin, Xianzheng Zhang

**Affiliations:** ^1^ Key Laboratory of Systems Bioengineering (Ministry of Education), Frontiers Science Center for Synthetic Biology, School of Chemical Engineering and Technology, Tianjin University, Tianjin, China; ^2^ James Watt School of Engineering, University of Glasgow, Glasgow, United Kingdom; ^3^ Key Laboratory of Biomedical Polymers of Ministry of Education & Department of Chemistry, Wuhan University, Wuhan, China

**Keywords:** engineered probiotic, host-bacterial interaction, single-cell analysis, intestinal diseases, synthetic biology

Gut microbiota, known as an important “organ” of the human body, plays an important role in regulating the host immune response, repairing the intestinal barrier, and resisting pathogenic bacteria invasion. The imbalance of intestinal microbiota is closely related to digestive system diseases, accelerating the occurrence and development of inflammatory bowel disease (IBD), colorectal cancer (CRC), irritable bowel syndrome (IBS), acute or chronic radiation bowel disease, colonic constipation, diarrhoea and other intestinal diseases. Microecological therapy targeting the structure and function of gut microbiota has attracted extensive attention in the biomedical scientific community (Cani, 2018) . The network meta-analysis (NMA) conducted by Zhang et al. suggested that *B.coagulans* had prominent efficacy in treating IBS patients. Thus incorporating *B.coagulans* into a probiotic combination, or genetically engineering the strain to amplify its biological function may be potential routes to treat IBS. Lyu et al. highlighted the mechanisms of *SpA* by which the gut microbiota impact gut inflammation and trigger the immune responses and discussed the potential of probiotics being an adjunctive therapy for *SpA*. Hao et al. evaluated the efficacy of probiotics in combination with prebiotics to treat patients suffering from hypothyroidism complications with small intestinal bacterial overgrowth during the second trimester of pregnancy. Yin and Zhu’s systematic review on the meta-analysis of clinical trials suggested probiotics have potential value in the treatment of Parkinson’s disease (PD)-related constipation.

With the development of multi-omics technologies, the genetic and metabolic characteristics of the gut microbiota have been deeply explored to develop new therapeutic interventions for the host (Agrawal et al., 2022). Modelling the spatial interaction network of gut microbiota has been built to reveal the causal relationship between spatial variability and changes in health states (Cao et al., 2022). Intestinal homeostasis is maintained in a dynamic equilibrium by balancing the contribution of different players, including diet and drug use. Traditional Chinese medicine and natural products play an important role in this process. Gut microbiota act as important regulators in inflammation and metabolic disorders (Wang et al., 2021a), relying on microbial metabolites and their interactions with receptors on host cells to activate or inhibit signalling pathways (Wang et al., 2021b). Che et al. elucidated the mechanism of the bidirectional interaction between traditional Chinese medicine and intestinal flora, as well as repairing the intestinal mucosal barrier and protecting the barrier function through various modalities. Thus, multiple interventions based on the modulation of the gut microbiota or the use of specific prebiotics and probiotics might contribute to the design of microecological agents.

Isolating and identifying microbes that can interact with probiotics provides an important basis for evaluating the efficacy of probiotics and clarifying their mechanisms. Yin et al. developed a single-cell droplet approach to obtain the isolates of the beneficial gut bacteria, which complements culture-independent metagenomic investigations of living bacteria therapy. Moreover, emerging technologies, such as Raman spectroscopy, flow cytometry and microfluidic technologies, have provided powerful tools to study microbiome function at the single-cell level (Yuan et al., 2017) and sorting cells (McIlvenna et al., 2016; Lee et al., 2019; Lyu et al., 2020). Wee et al. showed the feasibility of Raman spectroscopy and flow cytometry for phenotypic studies in long-term antibiotic treatment or when investigating new antibiotic classes.

Engineered probiotics are the next generation of live biotherapeutics that have been modified to target specific diseases. In recent years, engineered probiotics served as live biotherapeutics have been continuously created due to the rapid development of synthetic biology (Ozdemir et al., 2018). When disease marker molecules were detected, probiotics were programmed to release therapeutic effectors such as SCFAs (Bai et al., 2020; Wang et al., 2022), 5-HT (Li et al.) and active ingredients from plant sources. In this way, engineered probiotics have been used to improve metabolic disorders, behavioral disorders and cancer efficacy (Gurbatri et al., 2022). In addition to bacteria and fungi, bacteriophage engineering promises to generate phage variants with unique properties for prophylactic and therapeutic applications (Kortright et al., 2019; Dhanoa et al.).Researchers are mining the key components of bacteriophages to build synthetic biological systems (Xu et al., 2020).

The artificial flora designed and synthesized with the concept of synthetic biology is expected to overcome the existing shortcomings and achieve high efficiency, precision and control of microecological therapy (Wang et al.). On the other side, researchers use material or chemical strategies to modify probiotics to achieve therapeutic efficacies for treating intestinal diseases (Song et al., 2022). Fecal Microbiota Transplantation (FMT) is one of the recommended treatments for recurrent *Clostridioides diffificile* infection, but endoscopy and available oral formulations still have several limitations in their preparation, storage, and administration. Aira et al. used microcrystalline cellulose as the main excipient to maintain the viability of gut microbiota for a long time.

In conclusion, this Research Topic showcases the emerging multidisciplinary approaches, including gene editing, single-cell technology, and faecal microbiota formulation, for engineering and evaluating probiotics as potential therapeutical agents to treat intestinal diseases. We hope that readers find these articles informative and look forward to an exciting future for engineered probiotics.

## Author contributions

HH, HY and XZ wrote the manuscript. Both authors read and approved the final manuscript.
